# Long-term follow-up of neonatal severe hyperparathyroidism: redefining calcium management

**DOI:** 10.20945/2359-4292-2026-0074

**Published:** 2026-07-03

**Authors:** Inês Meira, João Menino, Patrícia Ferreira, Joana Queirós, Diana Silva

**Affiliations:** 1 Serviço de Endocrinologia, Unidade Local de Saúde de São João, Porto, Portugal; 2 Faculdade de Medicina, Universidade do Porto, Porto, Portugal

**Keywords:** Hyperparathyroidism, receptors, calcium-sensing, parathyroidectomy, calcium homeostasis

## Abstract

Neonatalsevere hyperparathyroidism (NSHPT) is a rare, life-threatening disorder caused by biallelic inactivation of the *CASR* gene, resulting in severe hypercalcemia and markedly elevated parathyroid hormone (PTH) levels in early life. Although total parathyroidectomy is often curative, long-term calcium balance and treatment requirements remain poorly understood. We describe the 25-year follow-up of a woman with NSHPT due to a homozygous *CASR p.Arg680His* variant who underwent total parathyroidectomy with autotransplantation at 32 days of age. Despite initial normalization of calcium levels, graft failure led to permanent hypoparathyroidism requiring long-term calcium and active vitamin D supplementation. Over time, calcium and calcitriol requirements progressively decreased despite persistently undetectable PTH, with recurrent episodes of hypercalcemia requiring careful dose adjustments. This case represents one of the longest documented follow-ups of genetically confirmed homozygous *CASR*-related NSHPT. The progressive decline in calcium requirements reflects impaired renal calcium excretion and an altered calcium-PTH set point characteristic of *CASR* inactivation. These physiological adaptations challenge the conventional supplementation strategies and suggest that standard hypoparathyroidism guidelines - largely derived from acquired or autoimmune forms - may require cautious individualization in patients with homozygous *CASR* variants, given their distinct renal calcium handling and the possibility of lower urinary calcium excretion. Our findings reinforce the complexity of long-term management in NSHPT patients and illustrates how calcium requirements may change over time. Long-term follow-up cases such as this may contribute to a better understanding of the physiological mechanisms and inform future guideline development for this rare condition.

## INTRODUCTION

Neonatal severe hyperparathyroidism (NSHPT) is a rare, life-threatening condition caused by homozygous or compound heterozygous inactivating variants in the *CASR* gene, which encodes the calcium-sensing receptor (CaSR) (^1^). This receptor, expressed in parathyroid glands and renal tubules, regulates parathyroid hormone (PTH) secretion and renal calcium handling, thereby maintaining calcium homeostasis. Inactivating *CASR* variants raise the calcium-PTH set point, resulting in inappropriate PTH secretion and hypercalcemia despite elevated calcium levels (^1^-^3^).

We present the 25-year follow-up of a woman with a homozygous *p.Arg680His CASR* variant who underwent early parathyroidectomy for NSHPT. This case illustrates the unique long-term metabolic course and the evolving calcium requirements that challenge conventional supplementation strategies and current management guidelines.

## CASE PRESENTATION

A 25-year-old female is under long-term follow-up in our endocrinology department for permanent hypoparathyroidism after a total parathyroidectomy performed for NSHPT.

At 32 days of life, she presented with severe hypotonia, prostration, feeding refusal, and weight loss. Laboratory evaluation revealed marked hypercalcemia of 6.15 mmol/L (24.6 mg/dL; normal range [NR] 2.2-2.8 mmol/L or 8.5-10.5 mg/dL), hypophosphatemia of 0.73 mmol/L (2.25 mg/dL; NR 1.45-2.5 mmol/L or 4.49-7.74 mg/dL), and markedly elevated PTH levels of 89.26 pmol/L (842 ng/L; NR 1.6-6.9 pmol/L or 7-59 ng/L). She was initially treated with intravenous fluids, furosemide (3 mg/kg/day), and intramuscular calcitonin (8 mg/kg/day), with partial biochemical improvement.

Because of persistent hypercalcemia despite medical therapy, surgical intervention was pursued. Total parathyroidectomy with autotransplantation of one gland into the left deltoid was performed without complications. Histopathology revealed two parathyroid glands; however, the right superior parathyroid specimen consisted only of thymic tissue.

After a transient decline, serum calcium and PTH levels returned to elevated levels - 3.16 pmol/L (12.5 mg/dL) and 26.07 pmol/L (246 pg/mL), respectively - by postoperative day 17. A CT scan of the parathyroid region localized an additional parathyroid gland in the superior pole of the right thyroid lobe. Due to recurrent hypercalcemia, a second surgery (right hemithyroidectomy) was performed three months later, and histopathology confirmed the presence of parathyroid and thyroid tissue. This achieved biochemical remission of hyperparathyroidism.

Biochemical screening of both parents revealed features consistent with familial hypocalciuric hypercalcemia (**Table 1**), while screening of other family members was unremarkable. Genetic analysis identified a homozygous likely pathogenic variant in the *CASR* gene (c.2039 G>A; p.Arg680His) in the patient, with both parents confirmed as heterozygous carriers.

**Table 1:** Blood and urine test results of the child and her parents

	Serum calcium (mg/dL)	Serum phosphate (mg/dL)	PTH (pg/mL)	FECa (%)	TRP (%)
Child (32 days)	24.64	2.25	842	0.9	10
Father (27 years)	10.4		46.8	0.4	
Mother (26 years)	10.0		33.7	0.5	

Normal range for serum calcium is 2.1-2.6 mmol/L (8.5-10.5 mg/dL). Normal range for serum phosphate is 1.45-2.5 mmol/L (4.49-7.74 mg/dL). Normal range for parathyroid hormone (PTH) is 1.6-6.9 pmol/L (7-59 pg/mL). Fractional excretion of calcium (FECa) (%) was calculated as:. In CaSR inactivation, FECa is typically <1% despite hypercalcemia. Normal range for tubular reabsorption of phosphate (TRP) is 85-95%.

**Figure 1:**
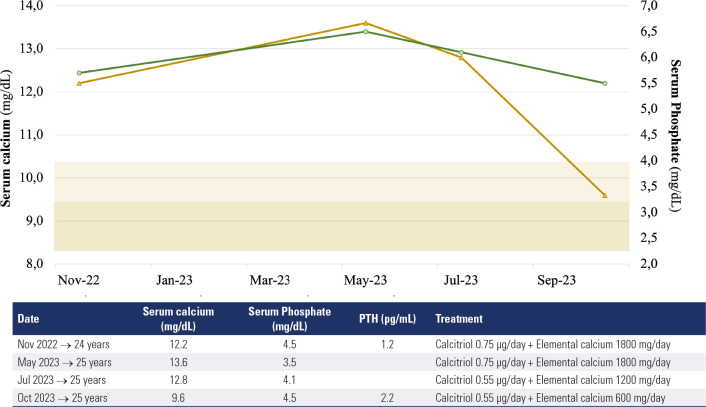
Serum calcium, phosphate, and parathyroid hormone concentrations over recent years and corresponding treatment. The yellow line depicts serum calcium values, while the green line represents serum phosphate values. The yellow box represents the normal calcium range, with the darker shade indicating the therapeutic target recommended in hypoparathyroidism guidelines (i.e., the lower half of the normal reference range or just below it).

The patient was discharged on vitamin D supplementation. However, due to autograft failure, she developed permanent hypoparathyroidism, requiring long-term treatment with calcium and calcitriol. She has remained under continuous endocrinology follow-up since childhood, with regular biochemical monitoring and treatment adjustments, and has been followed at our tertiary center since 2016. Over the past decade, repeated dose adjustments were required, and progressive reduction of calcium and calcitriol became necessary in the context of recurrent hypercalcemia (**Figure 1**). During this period, despite hypercalcemia, the patient consistently exhibited low fractional excretion of calcium. Specifically, at one point during follow-up, despite a serum calcium level of 12.6 mg/dL, 24-hour urinary calcium excretion was 12.8 mEq (approximately 256 mg), corresponding to a fractional excretion of calcium of approximately 1.4%, which is inappropriately low for the degree of hypercalcemia and supports impaired renal calcium excretion related to CaSR inactivation. Following treatment dose adjustment, with calcium carbonate 1500 mg/day (corresponding to 600 mg of elemental calcium), her serum calcium stabilized at 2.4 mmol/L (9.6 mg/dL). Following guideline recommendations to maintain calcium in the lower half of the reference range, calcium supplementation was temporarily suspended, leaving only low-dose calcitriol. However, the patient developed paresthesias and was unable to tolerate complete withdrawal of calcium, leading to the reintroduction of calcium carbonate at 1500 mg/day.

This case represents one of the longest documented follow-ups of genetically confirmed homozygous CASR-related NSHPT, highlighting the evolving calcium requirements over decades of care.

## DISCUSSION

The CaSR is a G protein-coupled receptor expressed primarily in parathyroid glands and renal tubules. By sensing extracellular calcium, it regulates PTH secretion and renal calcium excretion, thereby maintaining calcium homeostasis (^1^,^2^,^4^). More than 230 variants in the *CASR* gene have been described, leading to either lossor gain-of-function phenotypes (^3^). Inactivating variants raise the calcium-PTH set point, resulting in inappropriate PTH secretion and enhanced renal calcium reabsorption (^4^,^5^).

The clinical spectrum of *CASR* loss-of-function variants ranges from familial benign hypocalciuric hypercalcemia (FBHH), usually asymptomatic and caused by heterozygous variants, to NSHPT, a life-threatening disorder associated with homozygous or compound heterozygous variants (^3^,^6^,^7^). NSHPT typically presents in the neonatal period with severe hypercalcemia and markedly elevated PTH.

Initial management to control severe hypercalcemia includes intravenous fluid hydration and loop diuretics, with careful monitoring of volume status, as well as calcitonin, bisphosphonates, and calcimimetics (^8^-^10^). Although some reports describe neonates maintained on medical management (^11^-^13^), this approach is often complex and insufficient, and most patients ultimately require parathyroidectomy as definitive treatment (^14^,^15^). While subtotal parathyroidectomy was once preferred, most surgeons now favor total parathyroidectomy due to high recurrence rates (^15^). Autotransplantation of parathyroid tissue is commonly performed but carries a significant risk of graft-dependent hypercalcemia (≈33%) and a smaller risk of graft failure (≈6%) (^16^).

Postoperative permanent hypoparathyroidism is a frequent consequence following total parathyroidectomy without successful autotransplantation, and patients often require lifelong calcium and active vitamin D supplementation. Current guidelines for hypoparathyroidism recommend maintaining serum calcium in the lower half of the reference range or just below, to prevent hypercalciuria (^17^). However, our patient’s long-term evolution illustrates that this recommendation may not fully apply to homozygous *CASR* variant carriers.

Over the years, she required progressively smaller doses of calcium and calcitriol to maintain normocalcemia, despite undetectable PTH. This clinically counterintuitive finding can be explained by the reduced renal calcium excretion associated with *CASR* inactivation. Because the CaSR normally enhances urinary calcium loss in response to hypercalcemia, its dysfunction limits calciuresis and allows calcium balance to be maintained with minimal supplementation. However, this same mechanism also increases the risk of hypercalcemia if treatment is excessive (^2^).

Published reports describe heterogeneous biochemical and clinical outcomes after parathyroidectomy in patients with NSHPT. While some individuals remain dependent on lifelong calcium and active vitamin D supplementation (^10^,^18^,^19^), others can maintain normocalcemia with calcitriol monotherapy (^8^,^20^,^21^). This variability likely reflects underlying genotype-phenotype differences. Marx and Sinaii analyzed *CASR* genotypes, biochemical profiles, and clinical courses of patients with NSHPT, distinguishing two broad phenotypic patterns: (^1^) homozygotes for clearly pathogenic variants, characterized by severe neonatal hypercalcemia and very high PTH concentrations; and (^2^) compound heterozygotes, who often present milder neonatal hypercalcemia and demonstrate a greater likelihood of partial receptor activity. Interestingly, the authors observed that homozygous individuals, although initially more severely affected, frequently required lower doses of calcium and calcitriol after total parathyroidectomy to maintain normocalcemia (^22^). These findings suggest that even minimal CaSR signaling in peripheral tissues - particularly in the kidney - may persist and influence calcium homeostasis independently of parathyroid function.

From a physiological standpoint, the CaSR modulates renal calcium reabsorption primarily in the thick ascending limb and distal convoluted tubule (^2^). Inactivating variants reduce receptor sensitivity to extracellular calcium, thereby blunting the renal excretory response to hypercalcemia. In the postoperative setting, this translates into reduced urinary calcium losses and a diminished requirement for exogenous supplementation. In contrast, excessive calcium or calcitriol replacement can easily precipitate hypercalcemia, given the kidney’s impaired capacity to enhance calciuresis. This mechanism provides a plausible explanation for the wide interindividual variability in calcium needs and highlights the importance of cautious titration.

Furthermore, long-term tolerance to mild or moderate hypercalcemia in *CASR*-related hypoparathyroidism parallels that seen in FHH, in which heterozygous carriers remain asymptomatic despite chronic elevations of serum calcium (^1^,^3^). Similar to FHH, homozygous *CASR* variants carriers may exhibit lifelong protection against nephrocalcinosis and renal impairment due to persistently low urinary calcium excretion. Therefore, strict adherence to the conventional target of maintaining calcium in the lower half of the reference range may not be necessary (^17^) - and could even be counterproductive - in this specific population. Instead, maintaining biochemical stability and symptom control should take precedence over numerical targets.

In fact, current international guidelines for chronic hypoparathyroidism recommend targets that are derived from populations with acquired or autoimmune hypoparathyroidism and do not account for the unique renal physiology associated with *CASR* mutations.

Long-term calcium requirements in homozygous *CASR* mutation carriers remain unpredictable and may fluctuate over time. Some patients gradually reduce or discontinue supplementation as homeostatic adaptation occurs, whereas others relapse into symptomatic hypocalcemia after dose reductions. These divergent courses reinforce the need for personalized, physiology-based follow-up and careful long-term surveillance.

From a clinical perspective, this case illustrates that long-term calcium and active vitamin D requirements after parathyroidectomy in NSHPT may evolve over decades and differ substantially from those observed in other forms of hypoparathyroidism. Rather than rigid adherence to standard biochemical targets, clinicians should anticipate dynamic calcium requirements, closely monitor symptoms and laboratory parameters, and adjust supplementation cautiously to avoid both hypoand hypercalcemia.

Given the extreme rarity of NSHPT, evidence is confined to small cohorts and isolated reports, precluding formal treatment algorithms. Nonetheless, accumulating long-term follow-up data - as exemplified by our patient - are critical to refine management goals and to inform future guidelines. A shift toward a personalized, physiology-based approach, acknowledging the unique renal and systemic consequences of *CASR* inactivation, will likely improve clinical outcomes and optimize quality of life in this rare population.

## CONCLUSION

This case illustrates the complexity of long-term management in NSHPT due to homozygous *CASR* variants. Despite permanent hypoparathyroidism, our patient required progressively less supplementation, ultimately maintaining normocalcemia with minimal therapy. This clinically counterintuitive finding reflects impaired renal calcium excretion and suggests that, in selected patients with homozygous *CASR* variants, strict adherence to lower calcium targets may not always be necessary; however, any reduction in supplementation or acceptance of higher serum calcium levels should be individualized and accompanied by close biochemical monitoring.

Ongoing documentation of long-term outcomes in NSHPT cases will enhance treatment approaches and outcomes for this rare condition. Our case contributes to this limited body of evidence, emphasizing the need for individualized care and the development of guidelines adapted to CASR-related disorders.

## Data Availability

datasets related to this article will be avail-able upon request to the corresponding author.
